# Editorial: Chemokine receptors and signaling in wound healing

**DOI:** 10.3389/fphar.2023.1282233

**Published:** 2023-09-07

**Authors:** Zhenlong Chen, Richard D. Minshall

**Affiliations:** ^1^ Division of Endocrinology, Diabetes and Metabolism, Beth Israel Deaconess Medical Center, Harvard Medical School, Boston, MA, United States; ^2^ Departments of Anesthesiology and Pharmacology and Regenerative Medicine, College of Medicine, University of Illinois at Chicago, Chicago, IL, United States

**Keywords:** DFU, CCL28, synthetic polymer, stat3, atovaquone, burn injuries, ulcerative colitis

The aim of this Research Topic is to provide a forum for publications focusing on chemokine receptors and their signaling in wound healing (Chen et al.), (Apte et al.), as well as inflammation-related pathological conditions such as burn injury (Ono et al.) and ulcerative colitis (Manzanares et al.). Novel interventions targeting these diseases have been established by targeting cellular mechanisms and signal transduction cascades downstream of chemokine receptors, as described in the original articles and the review.

A wound is an injury that breaks the skin or other tissue in the body It is estimated that 1%–2% of the population will experience a chronic wound during their lifetime in developed countries, costing at least $28.1 billion annually for the Medicare population alone. The major cell types involved in cutaneous wound healing span from hematopoietic cells (such as blood platelets), immune regulators (such as neutrophils, T cells, dendritic cells, monocytes and macrophages), to structural cells (such as fibroblasts, endothelial cells, keratinocytes and epithelial cells). Interestingly, both chemokine receptors and their corresponding chemokine ligands are highly regulated in the cell types mentioned above during different stages of wound healing. Even though progress has been made in the role of chemokine receptors in chronic wound healing such as diabetic foot ulcers (DFUs), we are still far from the development of commercially available therapeutic strategies.

Diabetic patients have a 25% lifetime risk of developing DFUs, which precede amputation in up to 90% of cases. The underlying causes of DFUs are multifactorial, with impaired perfusion and chronic inflammation being important contributors. Neutralization of excessive chemokine CCL28 was proposed to accelerate wound healing in diabetic mice (Chen Z, 2023). In this article, the authors observed a higher level of CCL28 in plasma, skin and adipose tissue in both patients with type 2 diabetes mellitus and diabetic *db/db* mice. Furthermore, overexpression of adenovirus-CCL28 in dorsal skin of WT mice elevated levels of proinflammatory cytokines as well as CCR10 expression, while with reduced endothelial nitric oxide synthase (eNOS) expression. Topical application of neutralizing anti-CCL28 antibody (Ab) dose-dependently accelerated wound closure and enhanced levels of eNOS/nitric oxide (NO), the anti-inflammatory IL-4, and vessel density, while decreasing the level of IL-66 in *db/db* mouse wounds. The study suggests neutralizing CCL28 has potential as a therapeutic option for treatment of diabetic skin wounds such as DFUs.

In an elegant review (Apte et al.), the authors summarized current research on both natural and synthetic biomaterials and their effects on chemokine signaling in wound healing. Specifically, 28 chemokines and their signaling profiles and immune functions were identified in skin wound healing. The authors also summarized 4 natural (glycosaminoglycans, chitosan, alginate and silk fibroin) and 3 synthetic (polyethylene glycol, poly (lactic-co-glycolic acid and methacrylic acid)) biomaterials that are used to promote wound healing, along with their effects on chemokine signaling. Due to the complexities in the mechanism of action of biomaterials in the wound environment, for example, many chemokines share both pro-and anti-inflammatory properties and thus the dominant profile is considered a function of timing, many studies are done *in vitro* with isolated cells. Therefore, additional studies are needed to define the immunomodulatory effects of biomaterials on chemokine signaling, in particular with synthetic biomaterials.

Systemic inflammatory responses, such as elevation of IL-6 which is induced by thermal burn injury, can cause muscle wasting, a severe involuntary loss of skeletal muscle that adversely affects the survival and functional outcomes of these patients. The level of signal transducer and activator of transcription 3 (STAT3)—a downstream component of IL-6 signaling—is elevated with muscle wasting in various pro-catabolic conditions. Based on these findings, the authors (Ono et al.) proposed a novel mechanism for preventing thermal burn-induced skeletal muscle wasting. In a burn injury model in C57BL/6 mice, intraperitoneal injection of C188-9, a STAT3 inhibitor, reduced the activity of STAT3 and ubiquitin-proteasome proteolytic pathways, reversed skeletal muscle atrophy, and increased grip strength. Similarly, pretreatment of murine C2C12 myotubes *in vitro* with C188-9 reduced the same inflammatory and proteolytic pathways, suggesting that pharmacological inhibition of STAT3 signaling may be a novel therapeutic strategy for thermal burn injury.

Ulcerative colitis (UC) is a chronic relapsing disease characterized by diffuse mucosal inflammation, leading to accumulation of neutrophils in the colon. All current drugs for UC show limited success rates and potentially serious side effects. The authors (Manzanares et al.) tested whether atovaquone, a recent FDA-approved antimalarial drug, affects mucosal inflammation in UC. In a preclinical dextran sulfate sodium (DSS)-induced colitis model in C57BL/6J mice, atovaquone treatment promoted resolution of colitis by reducing neutrophil accumulation in the inflamed colonic mucosa. Further experiments indicated that atovaquone suppressed induction of CD11b expression in neutrophils, thereby reducing their polarization and migratory ability. These data suggest a novel role for atovaquone in attenuating neutrophil migration by promoting resolution of mucosal inflammation, highlighting the potential of this drug as a therapeutic strategy for UC.

Studies of cellular and molecular mechanisms, especially the downstream signaling elicited by chemokine receptors and the associated inflammation *in vivo* in animal models, *in vitro* with multiple cell types, as well as in human clinical samples, are fundamental for accelerating recent findings of the novel interventions for the diseases such as diabetic wound healing.

